# Spoilage of Chilled Fresh Meat Products during Storage: A Quantitative Analysis of Literature Data

**DOI:** 10.3390/microorganisms8081198

**Published:** 2020-08-06

**Authors:** Ngoc-Du Martin Luong, Louis Coroller, Monique Zagorec, Jeanne-Marie Membré, Sandrine Guillou

**Affiliations:** 1 SECALIM, INRAE, ONIRIS, Université Bretagne Loire, Route de Gachet, CS 40706, F-44307 Nantes, France; ngoc-du.luong@inrae.fr (N.-D.M.L.); monique.zagorec@oniris-nantes.fr (M.Z.); Jeanne-marie.membre@oniris-nantes.fr (J.-M.M.); 2 Université de Brest, Laboratoire Universitaire de Biodiversité et Ecologie Microbienne, UMT Alter’ix, F-29334 Quimper, France; louis.coroller@univ-brest.fr

**Keywords:** microbiological spoilage, modified atmosphere packaging, storage temperature, expert knowledge, text-mining, data analysis

## Abstract

A literature search was performed on spoilage of fresh meat products by combining keyword query, text mining and expert elicitation. From the 258 collected studies, a quantitative analysis was first performed to identify the methods which are the most used to evaluate spoilage beside the preservation strategies suggested. In a second step focusing on a subset of 24 publications providing quantitative data on spoilage occurrence time, associations between spoilage occurrence time of meat products and specific spoilage indicators were investigated. The analysis especially focused on factors well represented in the 24 publications, i.e., gas packaging (O_2_ and CO_2_) and storage temperature. Relationships between spoilage occurrence and several microbiological indicators were also sought. The results point out possible advantages of removing dioxygen in packaging to delay spoilage occurrence, whereas, in the presence of dioxygen, the carbon dioxide proportion in the gas mixtures was shown to influence spoilage occurrence. The collected data clearly reveal a potentially protective role of lactic acid bacteria. Besides, while a spoilage role could be attributed to *Pseudomonas* spp., the growth of mesophilic aerobic microbes, *Brochothrix* spp. and *Enterobacteriaceae* seemed independent of spoilage occurrence time.

## 1. Introduction

Meat products are an excellent source of valuable nutrients for human consumption, such as animal protein, phosphorus, zinc and iron [1,2]. The global consumption of meat products in 2015 was estimated at 41.3 kg per capita (bovine meat at 10.1 kg per capita, ovine and caprine meat at 2.1 kg per capita, pork meat at 15 kg per capita (excluding China) and poultry meat at 13.8 kg per capita) [3]. However, an important portion of meat and meat products are also lost or wasted every year. These annual losses represent approximately 20% of the initial meat production (304.2 million tons). These losses are essentially due to spoilage, characterized by a decrease in the sensory quality of the meat products [4]. There is a diversity of spoilage characteristics in meat products for consumers, such as changes in aspect (texture, slime or liquid production), various color deteriorations or off-odors [5,6]. These spoilage defects generally take place by different mechanisms. Meat spoilage can be caused by natural processes in meat, such as lipid oxidation [7,8] or autolytic enzymatic reactions in the muscle cells of the animal after slaughtering [9]. However, the major cause of spoilage is the unavoidable contamination by microorganisms (essentially bacteria) during the processing of animals into meat products [10] and their subsequent growth and metabolic activities during storage. Each processing step can influence microbial contamination, and storage conditions can shape the structure of bacterial communities, consequently impacting the occurrence of microbial spoilage over time [11].

Spoilage may shorten meat product shelf-life and lead to their rejection by consumers because quality expectations are not met [12]. Spoilage of products of animal origin has been recently reviewed [13]. In the latter paper, microorganisms associated with meat and dairy product spoilage are reviewed and factors influencing spoilage such as temperature, pH, salt and packaging atmosphere are described. However, the effects of factors are sometimes difficult to interpret since experimental conditions and food matrices may differ tremendously between studies. The aim of the present study was to collect studies through a rigorous procedure to perform a review of meat spoilage, in an ultimate objective to provide a quantitative analysis of the effects of selected processing factors on spoilage occurrence. First, studies were gathered by using both keyword search, expert elicitation and text mining. Second, from relevant selected studies, a quantitative analysis of the literature was performed on the methods generally used to monitor meat spoilage and preservation strategies suggested. At last, from a subset of publications providing quantitative data on spoilage over time, associations between spoilage occurrence time of meat products and specific spoilage indicators were investigated, highlighting the effects of storage temperature, dioxygen and carbon dioxide in Modified Atmosphere Packaging (MAP) on spoilage.

## 2. Materials and Methods

### 2.1. Query Process of Literature Data

Our bibliographic search aimed to collect relevant data associated with microbiological spoilage of meat products from scientific literature. A preliminary search from the Science Direct database, based on three main keywords (“meat” AND “microbiology” AND “spoilage”) in full-text, provided more than 8000 records. However, these records were mostly not relevant, since many of them were studies on pathogens in meat or in other food products (seafood, vegetables, etc.). Thus, an alternative procedure, combining expert opinion and data mining, was adopted for identifying the relevant keywords for search query functions.

**Figure 1:**
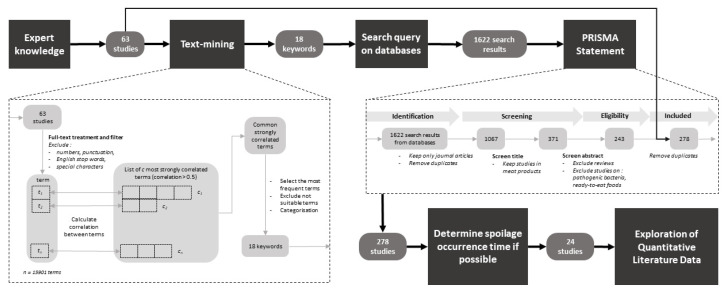
Flow chart outlining the procedure for quantitative data collection from the literature.

The procedure used to extract relevant keywords for query functions is detailed in Figure 1. First, 63 publications were initially provided by experts. Afterwards, the keyword extraction from the full text of these 63 publications was done using a text mining approach [14,15] using the tm R package [16]. This approach extracted the most relevant terms after different text treatment steps (filtering, categorization) based on correlations between terms, i.e., the occurrence frequency of multiple keywords in the same article [15]. The extraction was carried out following the procedure outlined by Williams (2016) [17]. The text mining of 63 publications provided an output of 18 relevant keywords. The data searches were carried out afterwards using advanced searches of full texts in two scientific platforms: Science Direct and Web of Science. While query functions required the “AND” function for the three principal keywords, (“meat”, “microbiology” AND “spoilage”), all supplementary extracted keywords were added with the “OR” option. The keyword and search function used were thus written as follows:

FULL-TEXT (meat * AND spoil * AND microbiology * AND (Brochothrix OR community OR data OR food OR lactate OR “lactic acid bacteria” OR “modified atmosphere packaging” OR odor OR psychrotroph * OR Pseudomonas OR volatile OR salt OR sodium OR refrigerated OR yeast)).

The latest query process using those above-mentioned keywords was performed on the 7 July 2019 and identified 1622 records. Afterwards, these records were filtered in accordance with the PRISMA Statement Guidelines [18] (Figure 1). Different criteria were used to exclude non-relevant publications after several stages of screening: (i) selecting from databases: only research journal articles were kept (book chapters, short communications and other article types were excluded) and duplicates from databases were removed; (ii) screening using titles: only studies on meat products in research articles were kept; (iii) screening using abstracts: review articles, studies exclusively on pathogenic bacteria in meat products and ready-to-eat foods were excluded; and (iv) removing duplicates already provided by experts.

After the screening using titles and abstracts, there were 243 research articles reporting microbiological spoilage in meat products, to which the 63 experts’ articles were added. After the removal of 20 duplicates, the remaining 278 articles were filtered, while removing reviews and studies specifically concerning modeling strategies, giving in total 258 articles. The overview of methods of spoilage characterization and preservation strategies was performed based upon these 258 publications. Then, in a more quantitative part, a subset of publications was performed to calculate what we called the spoilage occurrence time in selected publications providing quantitative data related to spoilage over time. In this respect, only studies in which sensorial evaluations were performed on meat products stored at temperatures between 0 and 12 °C were kept. Next, we removed studies in which the time interval between two sensory evaluations exceeded three days. This interval was arbitrarily chosen to approximate the determination of spoilage occurrence time with a maximum error margin of three days.

### 2.2. Collection of Quantitative Data Associated with Spoilage Occurrence in Meat Products

#### 2.2.1. Determination of Spoilage Occurrence Time

In each study, based on the characterization of organoleptic defects by scores (appearance, color, odor and flavor), the spoilage occurrence time was calculated as the time at which the considered scores exceeded a preliminary threshold value chosen by the authors. In cases where experimental sampling was carried out daily, we considered the observed spoilage occurrence time as the exact sampling time point. Otherwise, in cases where spoilage was observed after a two- or three-day interval between consecutive sampling times, spoilage was assumed to occur between the two sampling time points, and the spoilage occurrence time was considered as the median value of this interval (expressed in days).

#### 2.2.2. Factors Considered as Influencing Spoilage

In the 24 selected studies, a multifactorial design, i.e., combination of several factors, was followed. The considered factors were the type of meat products, storage temperature, initial gas composition (percentage of dioxygen, carbon dioxide and inert gases such as nitrogen or argon) of the packaging headspace (air, modified atmosphere packaging and vacuum-packaging). Hence, combinations of factor levels constituted different initial experimental conditions. For example: a study *i* on two types of meat products stored under three different modified atmospheres constituted six initial experimental conditions (*n_i_* = 6). In each study, the spoilage time was then estimated from multiple replicates, based on the criteria from sensory analyses detailed in the above section.

#### 2.2.3. Microbiological Data

Microbiological data associated with spoilage were collected from the selected studies. High throughput sequencing data were provided in some studies, but this type of information was not available often enough to be considered for the present study. Therefore, we considered only enumeration data acquired by conventional cultural methods for the five most studied microbial indicators, i.e., total mesophilic aerobic counts, lactic acid bacteria (LAB), *Brochothrix* spp., *Pseudomonas* spp. and *Enterobacteriaceae*. For each initial experimental condition, we determined the count of each microbial indicator observed at the spoilage occurrence time. If experimental sampling was carried out daily, we considered the bacterial count as the value observed at the sampling time point. Otherwise, if spoilage was observed after a two- or three-day interval between consecutive sampling times, the bacterial count at the spoilage occurrence time was considered as the average count observed at the two sampling time points.

The gathered dataset used for quantitative analyses included the following for each initial experimental condition: meat type, storage temperature (°C), initial composition of the atmosphere packaging (initial percentage of dioxygen and carbon dioxide), the corresponding spoilage occurrence time (days) and the bacterial count observed at the spoilage occurrence time (log CFU/g).

### 2.3. Statistical Analyses

The symmetrical Gaussian distribution of different studied variables, such as spoilage occurrence time, storage temperature or bacterial counts, were tested using the Shapiro–Wilk test. The evaluation of the difference between two groups following Gaussian distributions was performed using the Student’s t-test; otherwise, the non-parametric Wilcoxon rank sum test for non-Gaussian distributions was used. The difference between more than two groups was tested using ANOVA and Kruskal–Wallis rank sum tests for variables following Gaussian and non-Gaussian distributions, respectively. Correlation between variables was evaluated using non-parametric Spearman’s rank correlation test.

Pearson’s chi-squared tests were performed to evaluate differences between the observed and expected frequencies in contingency tables summarizing relationships between different variables. All statistical analyses were performed with the R program [19]. All graphs were created using the ggplot2 R package [20].

## 3. Results and Discussion

### 3.1. Measurement of Spoilage in Meat Products and Control Strategies

#### 3.1.1. Types of Meat and Meat Products Associated with Spoilage

As mentioned previously, the bibliographic search provided a set of 258 original research articles from 1984 to 2018 considered in our review on microbiological spoilage in meat products (Table S1). The most studied meat matrices corresponded to products from pork (117), beef (83) and poultry (54) (chicken or turkey) meats. The remaining meat matrices corresponded to lamb (21 studies), venison, goat, llama, foal or rabbit (fewer than five studies). Considering all meat matrices, the most studied meat types corresponded to fresh meats such as fillets, ground meats, strips, legs, carcasses, etc. (175 studies). Processed meats such as fresh sausages, dry or fermented sausages, mixed sausages, carpaccio, etc. were studied in 62 studies. Only 40 studied considered cooked meat products such as cooked ham or smoked sausages.

#### 3.1.2. Measurement of Spoilage in Meat Products

**Figure 2:**
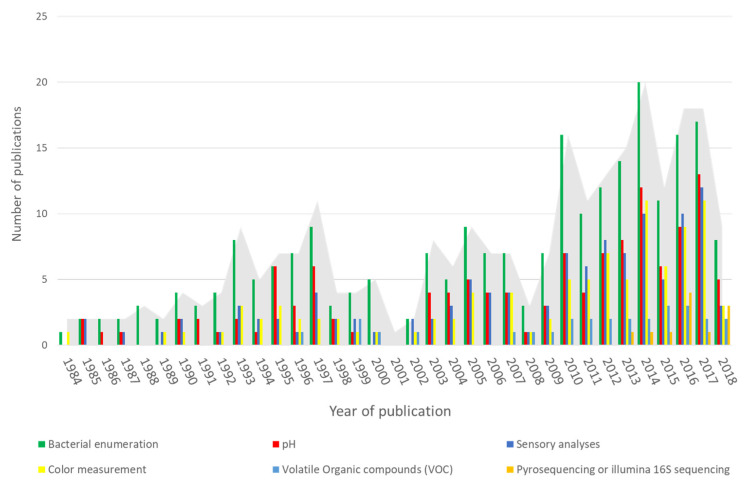
Publications considered in the review and evolution of approaches of spoilage assessment.

The exploration of all studies suggested a very important number of possible approaches for measuring spoilage. The most common methods of spoilage assessment were found to be classified in different categories: microbiological analysis, sensorial analysis and physicochemical analyses. Figure 2 illustrates the evolution of spoilage measurement among publications over the years.

It appears that microbiological analyses remained the preferred strategy of spoilage assessment over the years. It especially concerns enumeration of bacterial groups. In recent years, recent high-throughput technologies involving 16S DNA amplicon sequencing has emerged but regarding our dataset, it still corresponds to a limited number of publications. Besides, sensory analyses have always represented a large part of the responses studied while considering spoilage.

##### Microbiological Analysis

Enumeration of microbial groups by using conventional cultural methods on selective or nonselective agar plates, has constituted the unmissable method for assessing food spoilage. Bacteria were more largely enumerated than molds and yeast (46 studies). The most studied bacterial groups corresponded to Lactic Acid Bacteria (173 studies), total mesophilic aerobic bacteria (TAMB) (162 studies), *Enterobacteriaceae* (113 studies), *Pseudomonas* spp. counts (89 studies), *Brochothrix* spp. (81 studies) and coliforms (46). Psychrotrophic aerobic counts, total anaerobic counts, psychrophilic bacteria or *staphylococci* were less frequently monitored (between 19 and 46 studies), whereas other specific bacterial groups such as proteolytic bacteria (2), *Leuconostoc* (3), *enterococci* (8), H_2_S-producing bacteria (7), *Micrococcaceae* (6), or *Aeromonas hydrophila* (2) were scarcely monitored.

Isolate identification was performed in 70 studies plates mainly by genotyping methods such as molecular fingerprinting approaches using restriction enzymatic profiles such as Denaturing Gradient Gel Electrophoresis (DGGE); Restriction Fragment Length Polymorphism (RFLP) or ribotyping; or DNA amplification such as Random Amplified polymorphic DNA (RAPD) and Amplified Fragment Length Polymorphism (AFLP). These methods have been gradually replaced by sequencing of the 16S gene. Only two studies performed in 2016 and 2018 used mass spectrometry identification MALDI-TOF for isolate identification [21,22]. At last, characterizing microbiological communities in meat products has been nowadays facilitated by several emerging high-throughput sequencing techniques. Eleven studies from 2013 used this technology to assess semi-quantitively and identify the bacterial communities in meat products [22,23,24,25,26,27,28,29,30,31,32].

Phenotypic characteristics of isolate have been assessed in several studies either in pure culture or by re-inoculating the isolated strains in the products (41 studies). Apart from evaluating the organoleptic properties of inoculated products to confirm or invalidate the spoiler nature of the isolate, other more specific characteristics could be determined. Growth modeling was performed in 35 studies, mainly by using the Gompertz and Baranyi models. Acidification profiles of meat products were widely studied by pH monitoring or measurement of lactic acid [26,33]. Proteolytic, lipolytic or other potential metabolic activities assumed to be associated with a priori spoilage-responsible microorganisms were also measured in some studies [26,34]. Few studies also applied microbial Time Temperature Indicator (TTI) prototype for monitoring spoilage, based on the growth and metabolic activity of specific microorganisms [35,36]. The decomposition zone diameter (DZD) profiles of different microorganisms could be monitored to assess their potential spoilage ability [37]. Finally, some rare studies investigated other microbial abilities such as antibiotic resistance, salt tolerance, metabolite production (lactate, ethanol, glucose, etc.) as well as antibacterial activity towards pathogens [33,38].

##### Evaluation of the Organoleptic Quality

Based on our dataset, sensory analyses were identified as the most used approaches for evaluating the organoleptic quality of meat products (in 45% of all studies), probably because sensorial profiles are close to spoilage perception by consumers. In general, these approaches consist to determine the acceptability of food products by a panel constituted of several members. The number of members in the panel can vary from one study to another [39,40]. Depending on the types of meat (raw or cooked meats, red or white meats), the panel generally trained during preliminary sessions, determines different possible hedonic scales in order to evaluate organoleptic descriptors to be scored (intensity of off-odors or visible discoloration in fresh meats or general appearance as well as off-flavor for cooked products) [41,42]. A global score can be given afterwards to evaluate spoilage occurrence and intensity of the products using an acceptability rule, for example an arbitrary threshold value chosen by the panel in the used hedonic scale [43].

In addition to the use of sensory analyses, the organoleptic quality of meat products can also be evaluated using specific measurement instruments. For instance, we can cite the CIELAB color system which is widely used for measuring color of meat products [44,45,46,47]. Other measurements can also be carried out such as tenderness and texture (e.g., shear force) of beef steaks [48]; the weight loss during meat dry-aging, cooking or storage [49,50]; exudate volume in packages as well as package volume (blowing properties) [47,51,52]; or multispectral imaging assessment of meat surface for monitoring spoilage during storage [53].

##### Evaluation of Physicochemical Properties

The physicochemical response measured in half of studies is pH (Figure 2) since it reflects both bacterial growth and alteration of taste and flavor. Moreover, it is easy to measure and does not require any specific equipment. Activity water is more rarely measured (in 17 studies).

Other types of physicochemical responses can also be monitored in meat products in order to evaluate indirectly microbiological properties of meat products, assuming that microbiological spoilage results from metabolic activities of microorganisms. The most used responses corresponded to the identification and quantification of volatile organic compounds (VOCs) [54,55,56]. Volatile fingerprints from microbial activities in meat products could also be monitored using electronic noses for rapid assessment of meat quality [57,58]. Biogenic amines (BA) responsible for allergenic reactions were also sometimes monitored since their production is due to bacteria producing amino acid decarboxylases likely to induce meat spoilage [26,59,60]. Other chemical indicators for predicting the microbial quality of meat may also been measured even if it is less frequent, for example volatile amines by the total volatile basic nitrogen (TVB-N) or trimethylamine nitrogen (TMA-N) methods [61,62]. Gas composition in products packaging such as O_2_ and CO_2_ was measured in 38 studies since these gases could be absorbed or produced by microorganisms, and also because they are added in Modified Atmosphere Packaging (MAP) [63,64]. Myoglobin oxygenation and redox state, assessment of lipid oxidation by the TBARS method (Thiobarbituric acid reactive substances) are other methods that are used because they may be associated with organoleptic properties such as taste, odor, flavor or color.

#### 3.1.3. Preservation and Spoilage Control Strategies

##### Active and Modified Atmosphere Packaging

**Figure 3:**
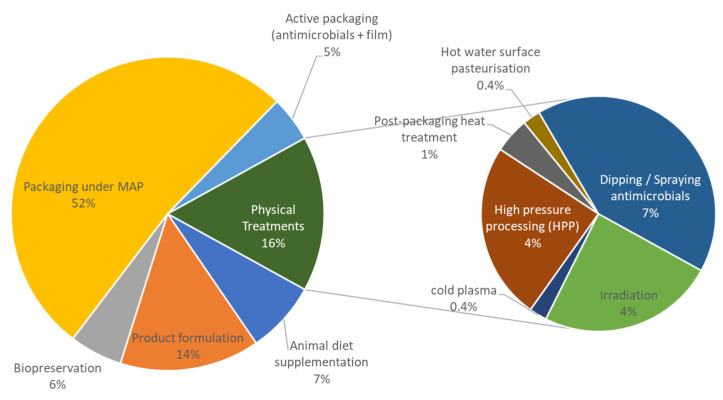
Main preservation strategies identified in collected studies.

Figure 3 illustrates the preservation strategies which have been studied in the collected dataset. The improvement in the characteristics of packaging for meat products were identified as the most common used strategies for preventing spoilage during storage. According to our dataset, in more than 50% of publications, meat products were conditioned under MAP. Vacuum packaging was often the other type of packaging under investigation. MAP consists in the replacement of the atmosphere surrounding the product by mixed gas compositions of O_2_, CO_2_, N_2_ and CO, as well as inert gases such as Ar. In general, the common gas compositions correspond to balances between O_2_ or CO_2_, since high O_2_ concentrations are useful for red meat color stabilization [65,66,67], and increasing CO_2_ in MAP is useful for inhibiting microbial growth [21,68]. In addition to MAP, some innovative methods of active packaging were also highlighted, either by injecting vapors of essential oils inside the package [69] or impregnating the film with essential oil [38,39,70,71,72,73,74,75,76]. The use of biodegradable nanocomposite packaging films containing TiO_2_ and rosemary essential oil [77], the adjustment of oxygen permeability for the films [78], the supercritical carbon dioxide (SC-CO_2_) treatment on the inactivation of the natural microbial growth [79] or chitosan/cyclodextrin treatment for packaging films [80] were also studied.

##### Formulation

Formulations of meat products was identified as a commonly studied strategy to prevent spoilage (used in 37 of 258 studies). Antimicrobial compounds susceptible to influence positively spoilage-related responses were used, mainly organic acids such as lactate, marinades, preservatives such as nitrites and essential oils. Many studies focused on the effects induced by lowering the concentration of food additives and/or replacing them by “clean” compounds. Some examples of formulation applications for preserving meat products are shown in Table 1.

##### Physical Treatments

Physical treatments corresponded to 16% of the studies, mainly the application of innovative treatments, such as High Pressure Processing (HPP) [95,101,102,103], treatments with aqueous ozone and electrolyzed water [104] as well as irradiation [105,106,107,108,109,110,111,112,113,114] and the use of cold plasma treatment [115]. Other treatments relative to addition of antimicrobials (urea, nisin, chitosan, oregano essential oil, lactate, etc.) by dipping or spraying the product were also studied as decontamination treatments or more generally as means of extending the shelf-life [41,43,116,117,118,119,120,121,122,123,124,125,126,127,128].

Other applications based on the influence of temperatures on spoilage have also been investigated such as rapid chilling, critical for meat hygiene, safety, shelf-life and nutritional quality [11,129], heat treatment at different processing steps [130] or hot water surface pasteurization [131].

##### Bio-Preservation

The use of controlled microbiota or antimicrobial flora for preserving meat during storage (principle of the bio-preservation) was reported in 14 studies [26,33,89,90,97,132,133,134,135,136,137,138,139,140]. These studies aimed generally to prevent spoilage occurrence or inhibit pathogenic bacteria to ensure safety by inoculating, during food processes, one or several microorganisms considered as protective cultures especially from LAB groups, or bacteriocins thereof. One can cite for example the use of *Lactobacillus rhamnosus* for inhibiting several strains of *L. monocytogenes* and *S. enterica* in fermented sausages [137], cultures of *Lactococcus lactis* for inhibiting pathogenic or spoilage spore-forming species such as *Bacillus cereus* or *Clostridium botulinum* in ham or Thai traditional fermented sausages [132,140], different mixed protective starters with *Lactobacillus sakei* for inhibiting *Staphylococcus carnosus* and *Staphylococcus xylosus* in dry fermented chicken sausages [97] or the use of *Lactobacillus plantarum* as efficient antimicrobial bio-preservatives on fresh chicken meat [139].

##### Animal Diet Supplementation

Some authors also studied the influence of the animal diet supplementation, especially in antioxidants in the improvement of the meat quality. These studies represented 7% of the collected data [47,141,142,143,144,145,146,147,148,149,150,151,152].

### 3.2. Spoilage Occurrence Time in Meat Products

#### 3.2.1. Spoilage Occurrence Time in Red and White Meat Products

**Figure 4:**
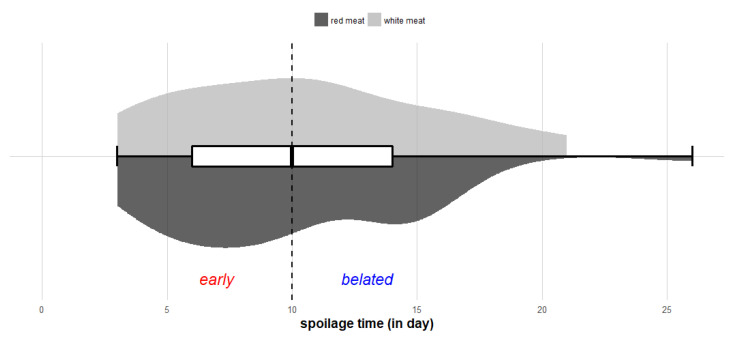
Distribution of spoilage occurrence time. The central boxplot represents the spoilage occurrence time obtained from all experimental conditions. The half violin plot represents spoilage time from studies involving red and white meat, respectively. The vertical dotted line represents the median spoilage time from all conditions, characterizing early and belated spoilage stages.

The second selection of publications providing quantitative data on spoilage over time resulted in 24 studies corresponding to data obtained from white (chicken or turkey) or red (beef, pork, ostrich, foal or lamb) meat stored under refrigerated temperatures between 0 and 12 °C. Several types of fresh products, such as raw fillets or fresh sausages, were included in the dataset (Table 2). In total, 84 initial experimental conditions (*N* = Σn_i_ = 84) were considered from the 24 chosen studies. The whole dataset obtained provided a global distribution of spoilage occurrence time, which varied from 3 to 26 days under refrigerated temperatures, with a median spoilage time estimated at nine days (Figure 4). Since this distribution was asymmetrical, the median time of nine days was defined as a critical threshold characterizing two principal spoilage stages. Spoilage occurring before the ninth day was considered “early spoilage” while that occurring from the ninth day onward was considered “belated spoilage”. Among the 41 experimental conditions showing an early spoilage stage, no spoilage was observed before three days.

Spoilage occurred between the third and sixth day in 24 experimental conditions and between the sixth and ninth day in 17 conditions. For conditions corresponding to belated spoilage, the spoilage occurred between the 9th and 16th day in 32 of 43 experiments. Thus, the spoilage occurrence time of fresh meat reached more than 16 days in 11 of 84 experiments under refrigerated temperatures, corresponding to about 13% of cases.

The above variability in the distribution of spoilage time led us to assume, firstly, that spoilage might depend on the type of meat products, i.e., red or white. To consider this, the distributions of spoilage occurrence time for red and white meat products were separately studied (Figure 4). In the case of white meat, the distribution followed a rather symmetrical Gaussian distribution and varied around an average value of 9.9 days. The average spoilage occurrence time in red meat products was slightly lower and estimated at 9.2 days. Although spoilage occurrence in red meats was mainly quite early, the belated spoilage could exceed 21 days and also had a slightly greater variability in comparison with white meat. Surprisingly, our data did not show any significant difference in spoilage occurrence time between red and white meats (*p* > 0.05—Wilcoxon test). This suggested that spoilage was observed at similar storage times in spite of the different characteristics of red and white meat products. However, the onset and kinetics of spoilage may depend on specific mechanisms, such as protein degradation or preferential metabolisms of spoilage-associated microorganisms, causing modification in color or off-odor [11,68,166].

#### 3.2.2. Influence of Initial Gas Composition in the Packaging

**Figure 5:**
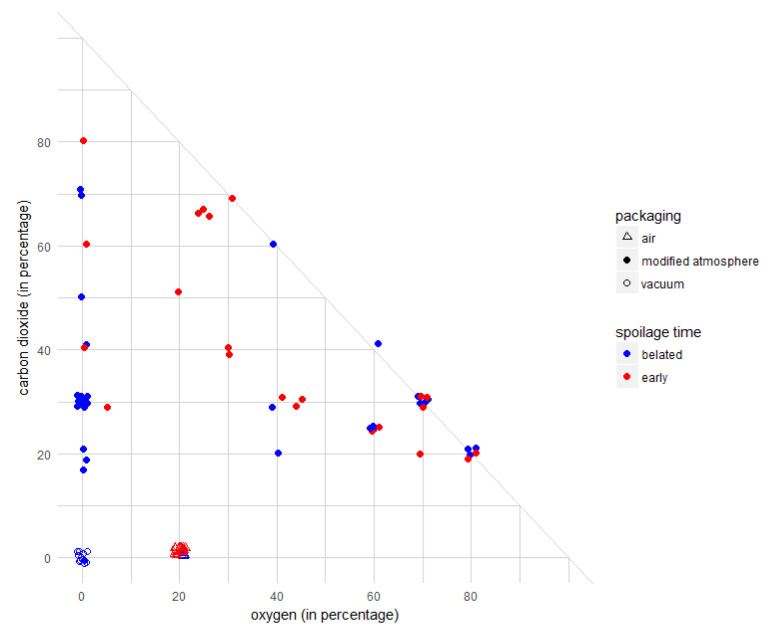
MAP triangular graph depicting the spoilage stage following different initial proportions of O_2_ and carbon dioxide in the packaging. The data are represented as jitter points to avoid overplotting. The blue and red symbols correspond to belated and early spoilage, respectively. The shapes of the points correspond to different types of packaging.

The dynamics of gas composition in the packaging headspace was not available for the 24 chosen studies; therefore, we focused only on initial compositions of the gas mixtures. Besides air or vacuum packaging in which no additional gas was added, several types of gas mixtures were used with different proportions of O_2_ and carbon dioxide, completed by inert gases (argon or nitrogen). The used gas mixtures were O_2_–CO_2_, O_2_–CO_2_–N_2_, O_2_–CO_2_–Ar, CO_2_–N_2_, CO_2_–Ar and 100% N_2_. The O_2_ and CO_2_ proportions varied from 5% to 80%. The distribution of the spoilage stages depending on the initial gas composition of O_2_ and CO_2_ and different types of packaging (vacuum, air and MAP) is represented in Figure 5. Plotting the spoilage stage as a function of the initial gas composition provided a global visualization of the effect of packaging. This graphical exploration enabled us to identify different visual patterns of spoilage, as well as advantageous options of MAP regarding spoilage delay.

Based on our data, removing O_2_ in the packaging seemed to be relevant for meat preservation. Indeed, spoilage under anaerobic packaging was generally delayed (Figure 5, blue points on the vertical axis). The packaging containing neither O_2_ nor CO_2_ represented MAP advantageous options of spoilage delay: the products were spoiled later in seven out of nine experiments. These conditions corresponded to vacuum-packaging or atmosphere under 100% N_2_ (rarely used, only in one study). Anaerobic MAP containing carbon dioxide (Figure 5) was observed as another MAP advantageous option of spoilage delay: spoilage was belated in 17 of 22 experiments. In all the above O_2_-depleted conditions, no significant difference in the spoilage occurrence time was observed between vacuum and anaerobic atmosphere packaging. Thus, regardless of the packaging mode and carbon dioxide concentration, O_2_ depletion appeared beneficial in terms of delaying spoilage occurrence.

Although spoilage occurrence was belated in most cases with anaerobic packaging, it was not possible to conclude with certainty that aerobic packaging accelerates spoilage. O_2_ was generally present with other gases. Indeed, gas mixtures with 60–80% O_2_ were used in 19 experiments (mostly for red meat products, such as raw pork and beef meat, probably to keep the red color). These compositions led to 7 early and 12 belated spoilages, and it is thus difficult to conclude the role of high O_2_ content. Conversely, packaging containing intermediate levels (from 20% to 50%) of O_2_ mostly led to early spoilage: 17 early spoilage events occurred in 20 cases under air and 10 events in 13 cases under MAP. Globally, regardless of O_2_ content, the spoilage occurrence time was shorter for products under air than under MAP. This difference might be explained by the higher initial concentration of CO_2_ in MAP. Finally, removing inert gases (Ar and N_2_) from initial aerobic atmosphere did not seem to impact spoilage occurrence time. Indeed, gas mixtures encompassing exclusively O_2_ and CO_2_ led to five early and nine belated spoilages (Figure 5).

**Figure 6:**
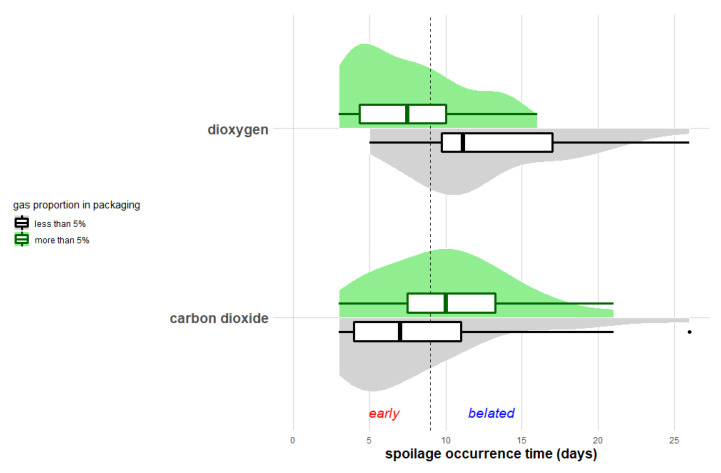
Spoilage occurrence time of meat products stored in packaging containing more or less than 5% of O_2_ or carbon dioxide. The vertical dotted line corresponds to the median spoilage time characterizing early and belated spoilage stages.

The above findings on various gas compositions (aerobic vs. anaerobic packaging and air vs. aerobic MAP completed with CO_2_) revealed implicitly separate effects of the presence of O_2_ and CO_2_ on spoilage occurrence time. Thus, we found it interesting to study the spoilage time according to the presence of these gases in packaging. A threshold value was then chosen in order to define the limit between what we considered the “presence” or “absence” of these gases in the packaging. In this study, this value was arbitrarily set at 5% of the gas proportion, since the minimal initial O_2_ percentage was 5% among the 84 experimental conditions. The distribution of spoilage time in the groups containing “less than” or “more than” 5% of O_2_ and CO_2_, are represented in Figure 6.

Spoilage occurrence varied differently in the presence and absence of O_2_ (Figure 6). In packaging with less than 5% O_2_, the spoilage occurrence time was rather distributed following a symmetrical distribution and varied around an average value of 12.7 days. In these almost anaerobic conditions, spoilage occurred mostly after the ninth day (in 25 of 32 cases). Conversely, with more than 5% O_2_, the spoilage occurrence time was asymmetrically distributed, with early occurrence in most cases, especially at the fourth day (Figure 6). Early spoilage corresponded to various gas mixtures, including packaging under air and other MAP, with different O_2_-CO_2_ proportions. Finally, the comparison of spoilage occurrence time between the “more than 5% O_2_” and the “less than 5% O_2_” groups highlighted a significant difference (Wilcoxon and Pearson’s chi-squared tests, *p* < 0.05). Therefore, the presence of O_2_ in packaging significantly impacted the spoilage occurrence time of fresh meat products.

The spoilage occurrence time seemed to also be impacted by the “more than 5%” or “less than 5%” carbon dioxide groups, since its distribution behaved differently in these conditions (Figure 6). In packaging with less than 5% carbon dioxide, the asymmetrical distribution indicated that there were more early spoiled products than belated ones. With less than 5% of carbon dioxide, most of the cases indicated early spoilage before the fifth day. These cases corresponded exclusively to packaging under air. The effect of CO_2_ on spoilage time was also evaluated in the same way as described above for O_2_. Surprisingly, our dataset did not show any significant statistical difference between packaging containing more than 5% or less than 5% CO_2_.

Spoilage was significantly delayed in the absence of O_2_; however, even under anaerobic conditions, the great variability and symmetrical distribution of spoilage occurrence time suggested that O_2_ depletion from the packaging did not ensure a belated spoilage for all products. Otherwise, under packaging containing O_2_, spoilage time was globally shortened, but also influenced by CO_2_ percentage. Moreover, O_2_ and CO_2_ concentrations in packaging can vary over storage time, which can differentially impact bacterial metabolic activities and meat spoilage processes during storage [68].

#### 3.2.3. Influence of Chilled Storage Temperature

**Figure 7:**
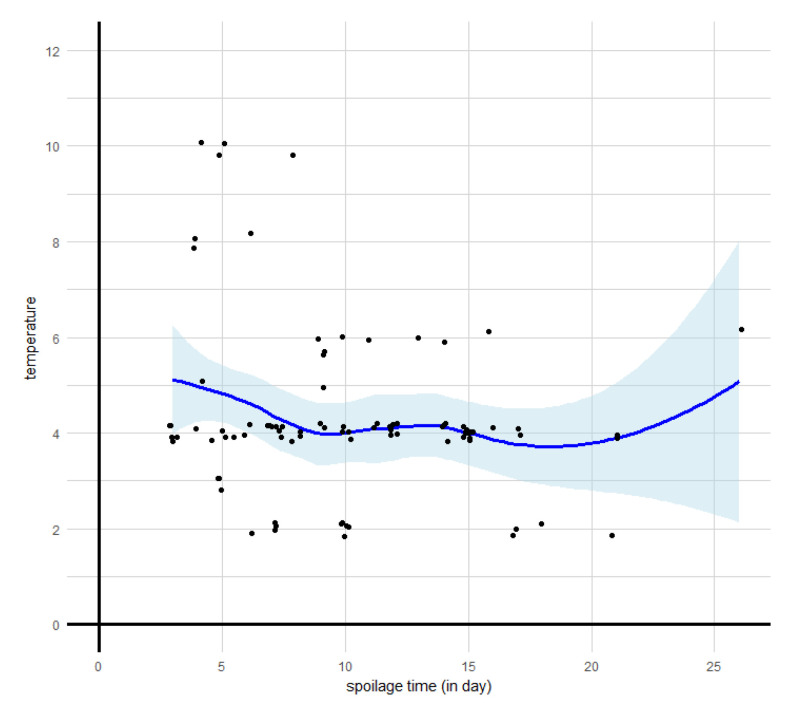
Spoilage occurrence time observed at different storage temperatures. The data are represented as jitter points to avoid overplotting. The thick black line represents the smooth curve obtained by LOESS local non-linear regression. The grey band corresponds to the 95% confidence interval around the smooth curve. The vertical dotted line corresponds to the median spoilage time characterizing early and belated spoilage stages.

The relationship between storage temperature and spoilage occurrence time is represented in Figure 7. Most experiments under refrigerated temperatures (between 0 and 12 °C) were carried out at 4 °C. The local regression showed a slight decrease of the smooth curve for early spoiled products (before the ninth day) and a stabilization phase at the belated stage (after nine days, inclusively). This trend could be explained by the fact that all products stored at higher than 8 °C spoiled very early. All products stored at temperatures between 2 and 6 °C did not necessarily spoil after our critical threshold of nine days. Indeed, in the case of products stored at 4 °C, the spoilage occurrence time showed great variability from 3 to 21 days. However, despite the observed trend from local regression, our data on refrigerated storage conditions did not enable us to highlight a significant correlation between temperature and spoilage time.

In this study, the difficulties in analyzing effects of storage temperature on spoilage occurrence time might be mainly due to several statistical reasons. Since data were collected from experiments performed under different MAP, with various types of meat, temperatures, etc., the data analyses are thus multifactorial. Although effects of temperature on microbial growth in meat products are incontestable [167,168], the multifactorial data analyses may have allowed the effects of storage temperature to be covered up by other factors. Moreover, as seen in Figure 5, very few data points corresponding to a spoilage time greater than 15–16 days were reported, which led to a bad estimation of the confidence interval. Finally, since most experiments were conducted at 4 °C, the non-Gaussian distribution of storage temperature led us to assess correlation between temperature and spoilage occurrence time using the inevitably appropriate non-parametric test, which usually has less statistical power than its parametric equivalent [169].

#### 3.2.4. Relationship between Spoilage Time and Microbiological Indicators

**Figure 8:**
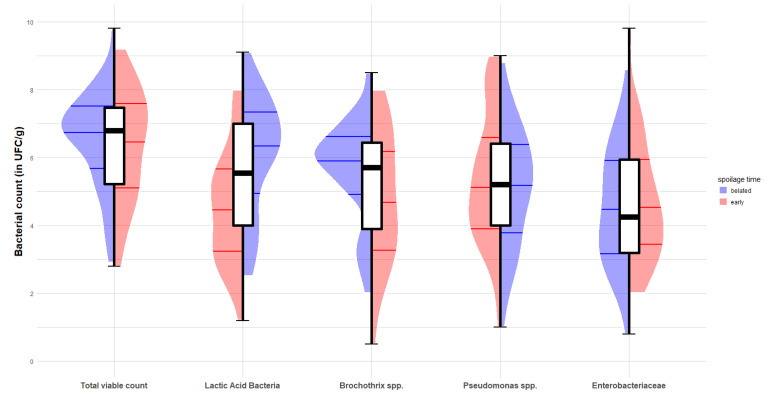
Distribution of bacterial count observed at spoilage occurrence time for different microbiological indicators in early and belated spoilage stages. The different plots correspond to different indicators. For each indicator, the central boxplot represents the overall distribution of the bacterial count. Half violin plots in red (or blue) represent the distribution of the bacterial count at the spoilage occurrence time in the cases of early (or belated) spoilage. Horizontal thick lines on half violin plots correspond to the first quartile, median value and third quartile of each distribution, respectively.

Five principal microbiological indicators were considered, i.e., total mesophilic aerobic counts, LAB, *Brochothrix* spp., *Pseudomonas* spp. and *Enterobacteriaceae*. The CFU enumerations are summarized in Table 2. The distribution of the counts for each indicator is represented as a function of the spoilage stage (Figure 8).

At the spoilage time and regardless of spoilage stage (early or belated), total mesophilic aerobic counts were observed as the most abundant group (6.52 log CFU/g in average) and *Enterobacteriaceae* as the least abundant group (4.61 log CFU/g in average). However, *Enterobacteriaceae* counts presented the highest variability range in comparison with the other groups, as their values ranged from 0.8 to 9.8 log CFU/g. Mesophilic aerobic counts were observed as the most abundant group, but the least variable, as its distribution varied between 2.8 and 9.8 log CFU/g with the lowest standard deviation. The remaining bacterial indicators, including LAB, *Brochothrix* spp. and *Pseudomonas* spp., had overall average and standard deviation values of approximately the same level. Globally, the results from the whole dataset (84 initial experimental experiments) showed a significant difference in the bacterial counts of the five bacterial indicators observed at the spoilage occurrence time, regardless of the spoilage stage (*p* < 0.05, ANOVA).

The comparison between early and belated spoilage suggested different distributions of the bacterial indicators in the two spoilage stages (Table 3 and Figure 8). Firstly, the distribution of total mesophilic aerobic counts was similar in early and belated spoiled products. The average counts were estimated, respectively, at 6.31 and 6.71 log CFU/g in early and belated spoilage stages. These results suggest that, regardless of the time at which spoilage occurred (before or after the ninth day), total mesophilic aerobic counts had grown in the same way as before spoilage. Hence, a low total mesophilic aerobic count may not necessarily trigger belated spoilage outcomes. The similarities in bacterial counts between early and belated spoilages were also observed for the groups of *Brochothrix* spp., *Pseudomonas* spp. or *Enterobacteriaceae*. These indicators seemed independent of the spoilage stage. In contrast, LAB counts in the belated spoiled products was significantly higher than in the early ones. The observed average values of the LAB count in spoiled products before and after the critical threshold of nine days were, respectively, 4.46 and 6.05 log CFU/g, with more than 3 log CFU/g of variability.

**Figure 9:**
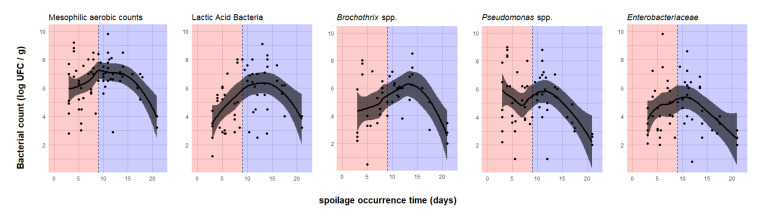
Enumeration of different microbiological indicators observed at spoilage occurrence times. The dotted vertical line corresponds to the median spoilage occurrence time characterizing early and belated spoilage stages, which are represented in red and blue backgrounds, respectively. The thick black curve represents the smooth curve obtained by LOESS local non-linear regression. The grey band corresponds to the 95% confidence interval around the smooth curve.

The results suggest that relationships between observed counts and spoilage occurrence time might differ from one bacterial indicator to another. In other words, all the studied groups do not grow in the same way before the occurrence of spoilage, so they may contribute differently to the spoilage process. To investigate this, for each indicator the correlation between their population level and the spoilage occurrence time was assessed (Table 3 and Figure 9).

For all indicators, spoilage occurrence after the 15th day was exceptional and associated with particularly low bacterial counts. In these conditions, the smooth curves and confidence intervals showed a decreasing trend, which corresponded to 11 experimental conditions from six different studies, mostly concerning raw chicken meat packed under anaerobic MAP or vacuum. Moreover, in two of the six studies, in addition to the use of MAP, meat products were also treated with different combinations of additives, such as oregano, rosemary oil or chitosan. These additives probably contributed to the exceptionally low bacterial population.

Considering all products spoiled within 15 days, the non-linear regression shown in Figure 9 suggested a clear ascending trend for the LAB and *Brochothrix* spp. count following spoilage occurrence time, a quite steady trend for the total mesophilic aerobic counts and *Enterobacteriaceae* and a slight decrease before the ninth day for the *Pseudomonas* spp. group. For LAB, a significant positive correlation was observed between the bacterial counts and the spoilage time. This correlation and the observed difference in LAB counts between early and belated spoiled products may, therefore, confirm a potential protective role of this group. Concerning *Brochothrix* spp., no significant statistical correlation between counts and spoilage time was observed. Figure 9 shows a confidence interval estimated much wider on the left side for this group, which means that the available data for *Brochothrix* spp. in early spoiled products are probably insufficient to describe correlation. For total mesophilic aerobic counts and *Enterobacteriaceae*, a steady regression curve and non-significant correlation coefficients (close to 0) were observed between counts and the spoilage occurrence time. Therefore, the development of mesophilic aerobic counts, *Brochothrix* spp. and *Enterobacteriaceae* seemed to be independent of the spoilage time. Mesophilic aerobic counts encompass a very diverse population. This bacterial indicator is obviously not specific enough to find correlation between counts and spoilage occurrence. For *Pseudomonas* spp., a softly decreasing trend and a slight negative correlation between counts and the spoilage occurrence time supports the assumption of the potential spoiling abilities of these bacteria.

## 4. Conclusions

The first step of the analysis highlighted the main responses which are generally monitored for spoilage evaluation and preservation strategies suggested. Among 258 studies, only 24 publications provided quantitative data enabling a quantitative analysis of the effects of gas packaging on spoilage occurrence time. It highlights that, in this field, studies are often descriptive or provide end-point characterization of spoilage which does not enable to perform kinetic analysis. Our results give a large overview of the relationships between spoilage occurrence and various indicators. The role of each bacterial group revealed from this study seems consistent with prior knowledge in the literature. However, our analyses were mostly based on cultural enumeration data, since the data issued from literature using high throughput sequencing, which is more informative about the bacterial groups (species or genera), unfortunately reported only bacterial relative abundance. The text-mining approach was also useful to identify relevant literature data, since it identified relevant keywords from articles provided by experts, thus enabling better targeting of further searches on scientific databases. Nevertheless, the quantitative analysis of spoilage-related data was, unfortunately, limited by the data available from research articles. Although the searches were performed on full texts to avoid omission of important studies, the thorough “manual” filtering and required selection criterion regarding the quantitative sensorial data over time led to the ultimate selection of only 24 studies to use for quantitative data analyses. The identification of influencing factors on spoilage occurrence time was consequently performed from quite a small dataset (84 initial experimental conditions in multifactorial dataset). The effects of these factors could not all be evaluated by hypothesis testing because of statistical significance problems. Indeed, using a multitude of hypothesis tests (and *p* values) on the same dataset may lead to uninterpretable test statistics, increasing the “false positive” effect and otherwise covering up the effects of other factors [170,171]. Therefore, for some factors, we preferred avoiding the hypothesis significance testing and using only graph interpretation instead.

We aimed to study the effects of several biological and physicochemical factors on spoilage occurrence time of meat products using a novel method for analyzing literature data. This method of combining experts’ opinions and the text mining approach for extracting relevant information enabled us to give an overview of multiple different studies. It also pointed out the deficit of quantitative data in research articles, which would be beneficial to dispose for further thorough studies relative to spoilage. Within the food microbiology frameworks, such methods could be profitably implemented, for instance for other food products, for which existing data are also available.

## Figures and Tables

**Table 1:** Application of formulation for preserving fresh meat and processed meat products.

Formulations	Meat Products	Observed Effects	References
Oregano essential oil	Chicken liver meat	Maintenance of freshness and sensorial quality, limitation of lipid oxidation	[81]
Cinnamon essential oil	Pork meat	Increase of microbial shelf life but unacceptable discoloration in high-oxygen atmosphere	[82]
Rosemary extract or oil	Fresh pork sausages	In combination with chitosan, extension of shelf-life of meat products; inhibition of lipid oxidation and rancidity	[83]
Parsley extract	Mortadella-type sausages	Inhibition of L. monocytogenes; improvement of overall appearance (color, cohesiveness, taste, aroma and saltiness)	[63]
Microencapsulated jabuticaba extract (MJE)	Fresh sausages	Natural dye used in replacement of commercial while maintaining antioxidant and antimicrobial activity and sensory acceptance	[84]
Satureja montana L. essential oil	Mortadella-type sausages	Antioxidant activity	[85]
Mixed spices and marinade (various ingredients and preservatives)	Beef (minced meats or steaks; pork meat	Alteration of microbial counts due to preservative addition with decrease of microbial diversity dominated by L. algidus and Leuconostoc sp. irrespective of the preservative tested. Glucose and packaging under oxygen in favor of spoilage.	[25,86]
Chitosan	Pork (loins, burgers, sausages); turkey and chicken breasts	Extension of shelf-life of low-sulphite burgers and turkey fillets, with synergistic effect on low dose sulphite or rosemary oil on spoilage prevention.	[41,87]
Sodium nitrite	Minced beef	Combined with essential oil stabilization of red meat color even at low dose; inhibitory effect on bacterial growth, control of lipid oxidation.	[85,88]
NaCl	Pork (loins, ground meat, ham, sausages)	Acid and exudate production following salt reduction, combination of MAP and low salt concentration correlated with sulfurous off-odors and higher spoilage than under vacuum, Maintenance of bacterial richness, inhibition of pathogens. Inhibition of growth of Aeromonas hydrophila at 3%.	[27,89,90,91]
EDTA	Beefsteaks; chicken (breasts, liver meat)	Combined to oregano essential oil and MAP extended the shelf-life of fresh chicken liver.	[81]
Lysozyme	Synthetic media (target products: processed ham and bologna)	B. thermosphacta inhibited by 500 mg/l or less lysozyme. Lysozyme also effective against P. acidilactici, En. faecalis and W. viridescens.	[89]
Vinegar, acetic acid	Pork sausages, fresh pork	Bacteriostatic properties of vinegar/sodium lactate mixture with reduced bacterial growth. CO_2_, citric acid and acetic acid reduced total growth.	[92,93]
Lactate	Pork sausages, pork meat, chicken fermented sausages	Lactate-diacetate altered the dynamics dramatically, yielding growth of a single species of Lactobacillus (L. graminis). Psychrotrophic, coliform and lactic acid bacteria retarded by lactate. No effect on sensory properties. Shelf-life extension. Synergistic effect between lactate and carbon dioxide. No or slight effect on color. Reduced effect with increased level of fat.	[24,94,95,96,97,98,99]
Polydextrose/glucose supplement	Hot-boned, mixed hindquarter cuts; minced beef	Increase of functionality properties of batters made with pre-rigor salted mince with added Polydextrose@. Similar composition and bacterial numbers in mince supplemented with glucose. Glucose, glucose 6-phosphate and lactic acid consumed at slower rates by the flora under MAP than in air.	[88,100]

**Table 2:** Data collected on spoilage occurrence time estimated from different experimental conditions in 24 studies. The cases filled in light grey correspond to the studied bacterial indicators, those filled in dark grey correspond to the spoilage indicators, i.e., measurement of sensory responses. N, total number of experimental conditions from all studies; *n*, number of experimental conditions in each study; *r*, number of replicates for each experimental condition.

References	Author, Year	Experimental Conditions in Each Study (*N* = 93)			Studied Bacterial Indicators (Enumeration)					Spoilage Sensory Indicators				
		[Red/White] Meat	Packaging	Replicate (*n* × *r*)	Mesophilic Aerobic Counts	LAB	*Brochothrix* Spp.	*Enterobac-teriaceae*	*Pseudom-onas* Spp.	Texture	Color	Odor	Flavor	Exudate/Drip Loss
[77]	(Alizadeh Sani, 2017)	[R] Lamb—Raw meat	Air	2 × 1										
[153]	(Balamatsia, 2006)	[W] Chicken—Breast fillets	Air; MAP	2 × 3										
[61]	(Balamatsia, 2007)	[W] Chicken—Breast fillets	Air; MAP; VP	3 × 1										
[154]	(Capita, 2017)	[R] Ostrich—Steaks	MAP	8 × 8										
[155]	(Chouliara, 2007)	[W] Chicken—Breast fillets	Air; MAP	6 × 1										
[43]	(del Río, 2007)	[W] Chicken	Air	3 × 6										
[34]	Ercolini, 2010	[R/W] Beef, Pork, Chicken—Various	Air	1 × 1										
[81]	(Hasapidou, 2011)	[W] Chicken—Liver breasts	Air; MAP	6 × 1										
[156]	(Herbert, 2013)	[W] Chicken—Breast fillets	MAP	6 × 1										
[157]	(Jääskeläinen, 2013)	[R] Pork—Raw meat	MAP	1 × 1										
[158]	(Jääskeläinen, 2016)	[R] Beef—Raw meat	MAP; VP	2 × 3										
[159]	(Kapetanakou, 2014)	[R] Pork—Steaks	MAP	4 × 2										
[160]	(Liu, 2006 #4619)	[R] Pork—Legs	Air; MAP	6 × 3										
[44]	(Lorenzo, 2012)	[R] Foal—Steaks	Air; MAP; VP	4 × 3										
[161]	(Martinez, 2006)	[R] Pork—Forelegs	MAP	5 × 2										
[162]	(Miks-Krajnik, 2016)	[W] Chicken—Breast fillets	Air	1 × 3										
[163]	(Nieminen, 2016)	[R] Pork—Loin	MAP	4 × 2										
[124]	(Petrou, 2012)	[W] Chicken—Breast fillets	MAP	4 × 1										
[164]	(Rahkila, 2012)	[R] Pork—Various	MAP	3 × 1										
[165]	(Rossaint, 2015)	[W] Chicken—Breast fillets	MAP	2 × 2										
[25]	(Stoops, 2015)	[R] Beef—Raw meat	MAP	3 × 3										
[67]	(Tremonte, 2005)	[R] Pork	MAP	3 × 3										
[41]	(Vasilatos, 2013)	[W] Turkey	VP	4 × 1										
[37]	(Wang, 2017)	[W] Chicken	Air	3 × 1										

**Table 3:** Enumeration of different bacterial groups at the spoilage occurrence time (overall, early and belated spoilage). N, total number of experimental data collected from all studies.

	Mesophilic Aerobic Counts	Lactic Acid Bacteria	*Brochothrix* spp.	*Pseudomonas* spp.	*Enterobacteriaceae*
N	71	62	45	66	59
[min-max]	[2.8–9.8]	[1.2–9.1]	[0.5–8.5]	[1.0–9.0]	[0.8–9.8]
early	[2.8–9.2]	[1.2–8.0]	[0.5–8.0]	[1.0–9.0]	[2.0–9.8]
belated	[2.9–9.8]	[2.5–9.1]	[2.0–8.5]	[1.0–8.0]	[0.8–8.6]
Mean ± sd	6.52 ± 1.59	5.36 ± 1.89	5.18 ± 1.82	5.28 ± 1.89	4.61 ± 1.83
early	6.31 ± 1.70	4.46 ± 1.68	4.74 ± 2.08	5.43 ± 2.04	4.55 ± 1.90
belated	6.71 ± 1.43	6.05 ± 1.76	5.50 ± 1.56	5.13 ± 1.75	4.67 ± 1.80
Correlation with spoilage time	0.0279	0.3208 *	0.1042	−0.2046	−0.0762

* *p* < 0.05, non-parametric Spearman’s rank correlation test.

## Supplementary Materials

Supplementary materials can be found at https://www.mdpi.com/2076-2607/8/8/1198/s1, Table S1: Data table of the 258 publications, highlighting the main responses measured to assess spoilage and preservations technologies (excel file).
